# Long COVID-19: A Concept Analysis

**DOI:** 10.3390/idr17040090

**Published:** 2025-07-29

**Authors:** Sujata Srikanth, Jessica R. Boulos, Diana Ivankovic, Lucia Gonzales, Delphine Dean, Luigi Boccuto

**Affiliations:** 1 Center for Innovative Medical Devices and Sensors (REDDI Lab), Clemson University, Clemson, SC 29634, USA; finou@clemson.edu; 2 School of Nursing, College of Behavioral, Social and Health Sciences, Clemson University, Clemson, SC 29634, USA; divanko@clemson.edu (D.I.); luciag@clemson.edu (L.G.); lboccut@clemson.edu (L.B.); 3 Department of Bioengineering, Clemson University, Clemson, SC 29634, USA

**Keywords:** long COVID, concept analysis, post-acute COVID syndrome

## Abstract

**Background/Objectives**: In late 2019, the Severe Acute Respiratory Syndrome Coronavirus 2 (SARS-CoV-2) caused a pandemic called the ‘coronavirus disease 2019’ (COVID-19). After the acute SARS-CoV-2 infection, many individuals (up to 33%) complained of unexplained symptoms involving multiple organ systems and were diagnosed as having Long COVID-19 (LC-19). Currently, LC-19 is inadequately defined, requiring the formation of consistent diagnostic parameters to provide a foundation for ongoing and future studies of epidemiology, risk factors, clinical characteristics, and therapy. LC-19 represents a significant burden on multiple levels. The reduced ability of workers to return to work or compromised work efficiency has led to consequences at national, economic, and societal levels by increasing dependence on community services. On a personal scale, the isolation and helplessness caused by the disease and its subsequent impact on the patient’s mental health and quality of life are incalculable. **Methods**: In this paper, we used Walker and Avants’ eight-step approach to perform a concept analysis of the term “Long COVID-19” and define its impact across these parameters. **Results**: Using this methodology, we provide an improved definition of LC-19 by connecting the clinical symptomology with previously under-addressed factors, such as mental, psychological, economic, and social effects. This definition of LC-19 features can help improve diagnostic procedures and help plan relevant healthcare services. **Conclusions**: LC-19 represents a complex and pressing public health challenge with diverse symptomology, an unpredictable timeline, and complex pathophysiology. This concept analysis serves as a tool for improving LC-19 definition, but it remains a dynamic disease with evolving diagnostic and therapeutic approaches, requiring deeper investigation and understanding of its long-term effects.

## 1. Introduction

In late 2019, a new virus, termed the severe acute respiratory syndrome coronavirus 2 (SARS-CoV-2), emerged in China and spread all over the world, causing a pandemic that has since been termed “coronavirus disease 2019” (COVID-19) [1]. This pandemic led to a global health crisis, resulting in the loss of over 7 million lives. Contributing factors included delays in virus identification, limited understanding of symptoms and pathogenesis, lack of effective treatments, and the prolonged vaccine development and distribution process [2]. Additionally, longer testing turnaround times and a lack of education surrounding the virus further fueled transmission [3]. Governments worldwide responded with emergency policy changes to protect public health and preserve economic stability amid uncertainty [4]. On an individual level, daily habits were transformed, emphasizing public health precautions, such as “extensive hygiene protocol (e.g., regular washing of hands, avoidance of face-to-face interaction, etc.), social distancing, and wearing protective masks [5].”

COVID-19 patients have a wide range of symptoms, including fever and fatigue and manifestations involving the respiratory, gastrointestinal, and neurological systems [6]. Many symptoms correlate to “central nervous system (CNS) involvement, like taste and smell impairment, headache and dizziness, and nerve pain [7].” After infection, patients developed the illness 2 to 14 days later, and the wide variability in disease duration and symptomology (asymptomatic pneumonia to multiorgan failure) made diagnosis difficult until the implementation of COVID-19 diagnostic tests [8,9,10,11]. Given the spectrum of disease severity, recovery time of symptom resolution and ability to return to regular lives varied [12]. 

As the pandemic progressed, new variants with genetic changes appeared, leading to increased infectivity and the ability for vaccine breakthrough infections [13]. Concurrently, a new class of patients started appearing in hospitals, individuals who had recovered from the acute viral attack with no detectable virus but continued to suffer from unexplained residual or new symptoms months later [12,14]. Unlike most infectious diseases, where symptoms subside after pathogen clearance, these patients remained symptomatic despite negative lab tests, highlighting a novel post-viral condition [15]. These patients are now diagnosed with “Long-haul COVID-19, Long COVID-19, or post-acute sequelae of COVID-19 (PASC)” [16,17].

Many initial Long COVID-19 (LC-19) cases involved older patients or those with co-morbidities, who were not well-documented or considered insignificant due to their age, ethnicity, or sex. Therefore, their symptoms were deemed psychological or imaginary [18]. Over time, the appearance of more patients with symptoms not attributable to other reasons prompted deeper clinical scrutiny [12]. Common new long-term symptoms noted in these patients include asthenia, myalgia, cough, insomnia, anxiety, dyspnea, abnormal chest imaging and pulmonary function tests, and cardiovascular issues [18,19]. Cognitive dysfunction, termed “brain fog”, was also widely reported, involving impaired memory, reduced concentration, and slowed thought processing, alongside anosmia (loss of smell) and dysgeusia (altered taste) [20]. Evidence indicates a significant correlation between LC-19 diagnoses and pre-existing psychiatric disorders, potentially confounding case ascertainment and inflating prevalence estimates. Consequently, psychiatric co-morbidities should be rigorously accounted for as covariates or exclusionary criteria in diagnostic and epidemiological assessments [21].

As the study of pulmonary, cardiac, and neural complications of Long COVID-19 (LC-19) continues, the long-term morbidity remains unclear. Mateu et al. analyzed over a two-year period the prognosis of LC-19 patients and reported a recovery rate of only 7.6% among their cohort, limited to those with milder symptoms [22]. Evidence suggests that in some patients, a threshold level of viral antigens may trigger a prolonged adaptive immune response that persists well beyond the acute phase of the illness [23]. Similarly to other infectious diseases, LC-19 may develop with increased risk following a severe acute disease or with longer viral shedding time [24].

Additionally, research shows vaccination status strongly correlates with LC-19 [25]. The benefit offered by vaccination is observed across different types of vaccines, regimens, and even incomplete regimens, showing protection against the prevalence of LC-19 [26]. Cohort studies involving healthcare workers who experience high levels of occupational exposure to SARS-CoV-2 have demonstrated elevated titers of virus-specific antibodies following primary vaccination, with further augmentation observed after booster administration [27]. Notably, the principal protective benefit of vaccination does not lie solely in the reduction in breakthrough infections, but rather in the attenuation of disease severity and a significant decrease in COVID-19-related morbidity and mortality. Öztürk et al. in 2025 found an improvement in symptoms of vaccinated individuals, regardless of the vaccine type, compared to the non-vaccinated individuals [28]. However, there were higher recovery rates in those with the mRNA vaccine BNT162b2. The relationship between vaccination and comorbidities is multifaceted. While vaccination has been shown to reduce the incidence and burden of Long COVID, even among individuals with pre-existing conditions, comorbidities remain an independent risk factor influencing the persistence and severity of post-acute sequelae, as acknowledged by the Centers for Disease Control and Prevention [29]. Preventing initial infection remains the most effective strategy for mitigating the risk of Long COVID. Staying up to date with COVID-19 vaccinations, including recommended booster doses, constitutes the most reliable and evidence-based approach currently available for reducing the likelihood of infection and subsequent post-acute sequelae. Given the sheer number of global infections, even a small percentage of individuals with long-term symptoms is a significant public health challenge. Evidence suggests that 7% of US adults have had LC-19, leading to 44.8 million cases as of 2025 [30]. The resulting LC-19-associated functional deficits carry profound national, societal, and personal consequences

A critical consideration of LC-19 is that it was initially conceptualized as a singular disease entity. However, emerging research increasingly supports the notion that LC-19 constitutes a heterogeneous spectrum of post-COVID-19 conditions, characterized by diverse underlying pathophysiological mechanisms [31]. The defining feature across all cases is their temporal association with a previous SARS-CoV-2 infection. As scientific understanding of LC-19 deepens, a more nuanced approach to diagnosing, managing, and supporting LC-19 patients is essential to mitigating its lasting impact.

### Purpose of the Analysis 

The significance of any concept hinges on numerous factors within its field and beyond, and, therefore, is constantly evolving. Hence, analysis is needed for concepts without precise definitions and characterizations to support their validity within a field [32]. The need for a concept analysis of LC-19 is of utmost importance as the term has been subjective and poorly defined, leading to ambiguity in its use in diagnosis and healthcare systems. Diagnosis of LC-19 involves a complete assessment of presented symptoms and excluding other conditions or causes, as there are currently no standard uniform regulations [33]. However, due to the variability of LC-19, including clinical presentation, timeline, and reactivation of latent viruses, diagnosis guidelines are challenging for healthcare personnel [34]. The World Health Organization, using a three-round Delphi consensus method involving patients and clinicians, helped provide a case definition for long COVID and an agreement on symptoms of LC-19 [35]. Although this study helped resolve some of the unknowns around presentation and symptomology, a continued discussion will support future research and management of LC-19 patients. The number of positive cases has been primarily collected using Electronic Health Records. Still, since not all symptoms have diagnostic codes for LC-19, there is an underestimation in the number of cases. This issue is exacerbated by the variability in onset time, lack of clinical symptoms exclusive to the condition, and absence of a conclusive diagnostic test [33].

Therefore, continual efforts to create a clear definition and establish the LC-19 condition as a concept are needed to advocate for improved strategies for patients and healthcare personnel. As recommended by Berger et al., “collecting comprehensive, public, transparent, patient-centered, and multidomain clinical and epidemiological data” was a crucial agenda worldwide to aid in these efforts [36]. Further analysis of clinical data to follow up on epidemiological outcomes of LC-19 patients compared to their self-questionnaires could help reduce variability and bias in these results. Additionally, as LC-19 has a clear antecedent, this continual study will be an essential means to study it as a post-viral condition. We use the Walker-Avant method of concept analysis to provide progress toward defining LC-19 [37]. By exploring the concept of LC-19 through this method, a relevant and functional definition of the term will evolve, and the impact of this concept may be recognized as it applies to healthcare.

## 2. Materials and Methods

### 2.1. Study Design

This analysis aims to understand this hitherto abstract, multifaceted concept and use empirical, data-driven, health-based attributes and definitions. This paper works to systematically and logically develop the parameters and boundaries to understand the term and further study the relationships between its properties. These will help establish guidelines to aid physicians, caregivers, and other healthcare professionals. During this process, we will deconstruct the term, define it, and examine the main elements/components to better understand its application and pertinence in nursing practice. The Walker and Avant method of concept analysis was used to define and distinguish the term LC-19 to understand and delineate it from other similar terms [37]. The Walker and Avant eight-step process is a classic method used to analyze, clarify, and establish a challenging and complicated phenomenon as a concept. It includes the following steps: (1) selecting a concept; (2) determining the aim of analysis; (3) identifying all possible uses of the concept; (4) determining concept-defining attributes; (5) identifying a model case; (6) identifying a borderline case; (7) identifying antecedents and consequences of the concept; and (8) defining empirical referents of the concept.

### 2.2. Data Sources and Analysis

Comprehensive literature searches for the terms were performed in dictionaries and online at the websites of the Centers for Disease Control (CDC), World Health Organization (WHO), Journal of American Medical Association (JAMA), and the NCBI website PubMed, MEDLINE, Elton B. Stephens Company (EBSCO) Cumulative Index to Nursing and Allied Health Literature (CINAHL) Web of Science and Cochrane Library. The selected MeSH terms used for the Boolean search with the operator ‘OR’ were “Long COVID”, “Long COVID-19”, “PACS”, “Long hauler”, “Long-haul COVID”, “Post COVID-19” and “prolonged COVID” in the titles and abstracts. Articles included had inclusion criteria, namely being peer-reviewed, relevant, adults, English language, full text articles, dated between Jan 2021 and March 2025, to identify publications relevant to the stated purpose of the analysis according to the Preferred Reporting Items for Systematic reviews and Meta-Analyses extension for Scoping Reviews, PRISMA-ScR [38]. Exclusion criteria applied were duplications and those irrelevant to the analysis.

## 3. Results

**Figure 1:**
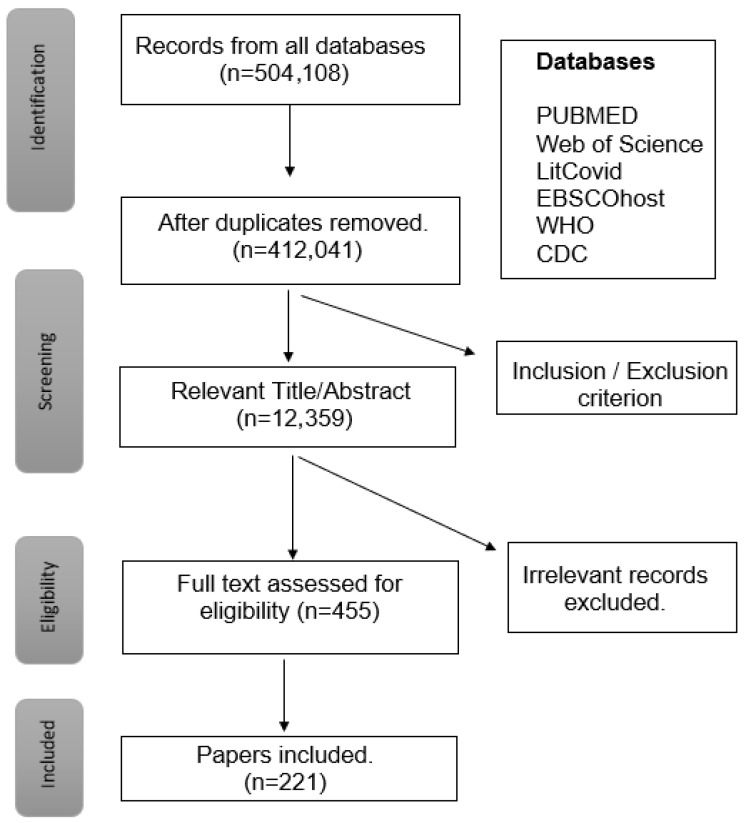
Preferred Reporting Items for Systematic Reviews and Meta-Analysis (PRISMA) flow diagram pathway to filter records from database output for inclusion within this study.

Papers were identified, screened, and included in the study based on the criteria presented in the flow diagram (Figure 1). The final 221 articles were reviewed and analyzed to assess the attributes, antecedents, and consequences of the LC-19 concept.

### 3.1. Dictionary Definitions of Long-COVID-19

The semantic meaning of the individual words in the term was assessed to aid in defining the meaning of the LC-19 concept. The terms comprising LC-19 were examined separately and together in several sources. The Oxford English Dictionary (OED) definitions of “Long” include “longitude as the greatest dimension of an object, to have a yearning desire or strong wish for something, to summon or send for (obsolete), to be a member or affiliate of a particular group or category (obsolete), and senses relating to duration (for example, a long time) [39].” As a suffix, it means “forming adverbs and (rarely) adjectives and prepositions indicating position or situation, for instance, sidelong [39].” The OED defines COVID-19 as an “acute disease in humans caused by a coronavirus, characterized mainly by fever and cough, and can progress to pneumonia, respiratory and renal failure, blood coagulation abnormalities, and death, especially in the elderly and patients with underlying health conditions [40].” Together, the two words would signify an extended version of acute disease, which is one of the definitions of LC-19.

Currently, LC-19 diagnosis includes symptoms that linger or appear after recovery from an initial COVID-19 infection [41]. Therefore, another definition is a medical condition suffered by patients who have had COVID-19 and continue to feel the effects up to weeks or months, involving many organ systems, even after a mild illness [14]. Symptoms of LC-19 can vary widely and include cough, low-grade fever, fatigue/malaise, chest pain, shortness of breath, headaches, cognitive dysfunction, anxiety/depression, chest/throat pain, and gastrointestinal upset (Table 1) [18].

The underlying cause of LC-19 is still a puzzle to researchers. Many theories have been proposed to explain the LC-19 symptomology. These include the viral persistence theory [47], autoimmunity [48], chronic inflammation [49], mitochondrial dysfunction [50], gut microbiome dysbiosis [51], latent viral activation [52] and dysregulation of cytokines [53]. Furthermore, patients with an LC-19 diagnosis typically have individual biological factors or co-morbidities that can contribute to long-term health concerns [54]. Co-morbidities like lung disease, cardiovascular disease, diabetes, kidney disease, mental illness, female sex, and persons aged ≥45 years increased risk [55]. Research has identified some genes associated with LC-19 symptoms to understand the variability of symptoms and begin efforts to define LC-19 (Table 2).

### 3.2. Defining Attributes

As the very name indicates, LC-19 is preceded by a positive COVID-19 test or at least an exposure to the virus [18]. A consensus was achieved regarding LC-19 by defining the most prevalent symptoms as “fatigue, headache, attention disorder, dyspnea, shortness of breath, cough, chest pain, muscle pain, joint pain, anosmia, dysgeusia, insomnia, anxiety, depression, concentration deficit, and mood changes [66].” Due to the recent pandemic and its longer-term sequelae, there is a lack of knowledge about the condition, further baffled by the seeming randomness and unpredictability of the symptoms. Distinguishing attributes of LC-19 span numerous sectors of the patient’s lives and their communities, including impacts of “limitations in health, financial status, social interactions and stigma [67].” Therefore, this multilayered impact of LC-19 can delay the patient’s path to recovery due to the increased burden associated.

One of the defining characteristics of LC-19 is its temporal profile, with persistent or newly emerging sequelae typically manifesting at least three months after the initial diagnosis of COVID-19 [18]. Infections with the Omicron variant resulted in earlier (4 weeks) LC-19 symptoms, although the actual numbers were comparable to the other variants by one year [68]. Respiratory failure, memory difficulties, kidney injury, mental health diagnoses, chronic fatigue, cough, cardiac and coagulation issues are all currently investigated to diagnose LC-19 [69]. Eponymously, the condition persists for a prolonged period with changes in the intensity of symptoms, involving numerous, sudden flare-ups of the indications. Studies of recovery from LC-19 are limited and inconsistent, primarily due to varying definitions and inadequate capture of the condition’s full spectrum. Research reveals recovery in those with fatigue as a primary LC-19 manifestation and symptomology (cardiovascular co-morbidities, hyporexia and anosmia/dysgeusia) during the acute phase. In contrast, those with muscle pain, impaired attention, dyspnea, or tachycardia, conversely, were less likely to recover [22]. Due to the recent nature of COVID-19 and, thus, LC-19, there is still insufficient information on the duration of the clinical manifestations and whether it will be a lifelong burden on the affected individual’s health [70].

A second attribute is the condition’s effect on the patient’s inability to resume his life to pre-infection standards, which may involve the ability to hold a job, leading to financial burdens. Patient-reported outcome measures (PROMs), including subjective diminished work capacity estimations, are frequently used as diagnostic indicators [71]. In most cases, post-COVID reductions in occupational functions cannot be corroborated by objective metrics such as the Work Ability Index (WAI), due to the absence of a baseline pre-infection. Recent simulations of the total cost of LC-19 indicate a $2.01–$6.56 billion cost to society in the US, primarily as productivity losses due to the inability to work [30]. Worldwide, the incidence of LC-19 is around 400 million, with an estimated economic impact of $1 trillion [72]. Current research indicates 30.2% of the participants had symptoms that impacted daily life [73].

A third attribute is the diminished quality of life and social interactions for individuals with LC-19 due to reduced work efficiency, decreased mobility, and continual symptoms [74]. Additionally, studies indicate that LC-19 impacts the patient both socially and emotionally, including a lack of confidence, uncertainty about the future, and elevated stress. Many individuals battle with fear, loneliness, and helplessness, leading to depression [75]. This impact is furthered when individuals are forced to take disability benefits, which adds a burden of shame.

### 3.3. Model, Borderline and Contrary Cases

As defined within the Walker and Avant guidelines, “a model case presents all the defining attributes of the condition described with a definitive example, a borderline case presents some, but not all, attributes, and a contrary case does not exhibit any of the characteristics of the concept being defined [37].

#### 3.3.1. Model Case

A 28-year-old previously healthy female was diagnosed with COVID-19 in August 2020. She arrived at the University outpatient clinic with a high fever, cough, myalgia, anosmia, and rash, symptoms suspected to be associated with COVID-19. She was discharged, advised to self-quarantine, and received customized care for the management of physical and psychological symptoms. During this period, her symptoms were very severe, but she managed despite a lack of better resources at her disposal at the time [76].

Two months later, she continued suffering from breathlessness when walking, fatigue, persistent fever, myalgia, frequent neurological manifestations including ‘brain fog,’ headache, cognitive impairment, and other symptoms. Therefore, she visited the accident and emergency services twice due to increasing concerns about her symptoms. She was diagnosed with persistent tachycardia and shortness of breath. After several investigations, including blood tests, ECG, and chest X-rays, she was discharged home with appropriate recommendations for domiciliary care. Over the next few weeks, her symptoms’ cumulative effect became so debilitating that she remained bedbound and unable to resume professional work.

Further assessment from her respiratory physician was performed due to concerns about potential persistent bronchial inflammation, disordered breathing, and other neurological symptoms. A follow-up assessment one month later showed no significant improvement in her clinical condition despite regular repeat investigations. As all the investigations and imaging recommended by the NICE guidelines could not explain the clinical condition, she was given the most plausible diagnosis, long COVID syndrome.

This model case shows all the defining attributes of LC-19, namely pulmonary issues, along with the other cardiovascular or neurological symptoms after a COVID-19 diagnosis. The patient was suffering at both physical and emotional levels, making her unable to work and continue her regular routine. LC-19 was completely debilitating her, causing a detrimental effect on her quality of life.

#### 3.3.2. Borderline Case

Building from an example case within the literature, a student (female, age 28, and Caucasian) had unexplained, debilitating symptoms, causing her to take leave from school and return home with her parents. Although athletic, she was “unable to perform any physical activity, and even daily functions such as showering would leave her exhausted (post-exertional malaise) [77].” Tests showed the patient’s vitals within a standard range [BMI: 20.2, BP: 97/51, pulse: 68, RR: 14, Temperature: 36.7 °C], but gastroenterology studies indicated impaired ability to break down easily digestible foods. She was COVID-19 negative and had never tested positive by the Polymerase Chain Reaction (PCR). Also, she tested negative for SARS-CoV-2 through antigen detection by immunohistochemistry and immunofluorescence for spike and nucleocapsid protein.

Over a period in 2022, as her symptoms persisted with increased fatigue and discomfort, she consulted “multiple physicians (urgent care, internal medicine, infectious disease, and family medicine).” She received a diagnosis of myalgic encephalomyelitis/chronic fatigue syndrome (ME/CFS) from the last physician utilizing CDC guidelines, which included “profound disabling fatigue for at least 6 months that remained unexplained and was accompanied by frequent sore throats, impaired cognitive function, post-exertional malaise, unrefreshing sleep, headaches, and joint/muscle pain [77].” Additional testing was performed for other diseases that could contribute to her symptoms. Epstein–Barr virus (EBV), a common human herpes virus that can cause mononucleosis, and an infection that leads to inflammation in the lungs, could be attributed to her severe myalgia and respiratory symptoms [78]. Autoimmune diseases like Systemic Lupus Erythematosus (SLE) or rheumatoid arthritis (RA) also give rise to similar symptoms [79,80]. However, following clinical testing, these conditions were ruled out.

Due to the unknown cause and expression of ME/CFS and the lack of a diagnostic test, criteria for ME/CFS diagnosis were developed through the consensus of experts. The International Consensus Criteria (ICC) describes ME diagnosis criteria with patients’ symptoms across numerous categories: “post-neuroimmune exhaustion, neurological impairment, immune/gastrointestinal/genitourinary impairment, and energy production/transportation impairment [81]. Historically, in diseases lacking a clear etiology and causative agent, criteria to aid diagnoses are defined based on the consensus of clinicians. Although this patient had similar symptoms to LC-19, such as fatigue and gastrointestinal issues, as she did not have a positive COVID-19 exposure, this case would be considered borderline within the LC-19 concept.

#### 3.3.3. Contrary Case

A contrary case example would be one of severe accidental hypothermia [82]. In late winter 2016, as outlined in the presented case, a 34-year-old female with a history of drug use and mental health issues was discovered to be missing for seven days before being found, having considerable exposure to sub-zero temperatures. Upon arrival at the hospital, the patient was unresponsive with a Glasgow Coma Scale (GCS) of 4, severely hypothermic with a rectal temperature of 23 °C, a heart rate of 30 beats per minute, a respiratory rate of 6 breaths per minute, and unmeasurable blood pressure and pulse oxygen saturation [82]. Cardiologic testing showed no detectable pulse and a dilated heart with a soft ascending aorta. After a heparin infusion, she was rewarmed to normal body temperature at a rate proper to improve survival and minimize neurological risk. To prevent hypothermia-induced coagulopathy, she was administered plasma and anticoagulants to decrease the risk of post-operative bleeding.

This contrary case indicates that the patient’s condition has none of the defining attributes of LC-19. The case was from a pre-COVID period, and the patient had no symptoms of pulmonary, cardiovascular, or neurological complications generally associated with the condition.

### 3.4. Antecedents

Antecedents are defined as events that happen before the diagnosis of the condition. In the case of LC-19, the primary antecedent is a COVID-19 infection [83], however, a positive COVID-19 test may not be necessary to diagnose a patient with LC-19 clinically. Individuals who were asymptomatic or lacked access to testing may not have confirmed test results but still experienced the infection [84]. In previously hospitalized COVID-19 patients, persistent ill health is commonly reported, with ongoing symptoms such as breathlessness, cough, fatigue, and mental health problems [85]. Even individuals with initially mild COVID-19 infections may experience unresolved symptoms, including persistent fatigue and breathlessness, headache, chest heaviness, muscle aches, and palpitations [14,86,87].

While not necessary antecedents for an LC-19 diagnosis, there are several risk factors that, when present before symptom onset, may contribute to a worse associated prognosis. These risk factors for developing LC-19 may become antecedents, including “belonging to the female sex, belonging to certain ethnic groups (Black Afro-Caribbean, Native American, Middle Eastern or Polynesian mixed ethnicity as compared to white ethnic groups), socioeconomic deprivation, smoking, obesity, certain prescription drugs, and other co-morbidities [54]. Additional biological antecedents may contribute to the chronic pathology of LC-19, which can include malfunction of the microbiota–gut–brain axis and reactivation of latent viruses, such as Epstein–Barr virus (EBV), cytomegalovirus (CMV), or human herpes virus 6 (HHV-6) [88].

Mental health conditions may also be an antecedent to LC-19. Individuals with a pre-existing history of depression have demonstrated a higher incidence of LC-19 diagnosis, lending support to the hypothesis that some manifestations of long COVID may be partially attributable to underlying psychological conditions [89]. Furthermore, a diagnosis of LC-19 can lead to the inability to perform regular daily routines and negatively affect the quality of life [90]. The decline results in consequences of social isolation, frustration, restriction of movements, and reduced everyday interactions with others in society [67]. Therefore, individuals with pre-existing mental health conditions who have already experienced such stressors may be more susceptible to increased LC-19 symptoms and prolonged recovery [91].

### 3.5. Consequences

Consequences are the events that follow the concept’s occurrence. Although LC-19 cases are present in a small proportion of COVID-19 cases, the sheer magnitude of the number of patients affected by the virus has made the post-viral functional deficits a significant burden [2,92]. On a national level, the compromised work efficiency has severely affected the economy; societal consequences are caused by the inability to earn a livelihood and dependence on community services [93]. Finally, the isolation and helplessness caused by the disease, with its subsequent impact on mental health and quality of life on a personal scale, are virtually immeasurable [94].

Many patients with LC-19 are unable to hold down their pre-COVID-19 jobs due to impaired health (documented by the clinician and Patient-Reported Outcome Measures (PROMs) [89]. This dependency may result in loss of employment and displacement from their homes, ultimately causing shame and humiliation. Such feelings by the head of the family have a trickle-down effect on the rest of the members, inducing helplessness and emotional constriction [95]. Studies on the impact of Long COVID on employment indicate that 7.2% of participants reported missing >10 workdays and 13.9% reported not returning to work [96]. The virus impacts survivors not only physically but also psychologically, and therefore, after COVID-19, and especially after a diagnosis of LC-19, it is essential to seek treatment for all changes in both body and mind [97]. LC-19 patients report mental health symptoms, such as depression, anxiety, posttraumatic stress disorder (PTSD), and insomnia, that could be worsened by pre-existing or newly developed conditions [75].

Intense studies on LC-19 and its associated pathogenicity, risk factors, and long-term societal effects have shown interesting and follow-up-worthy results because of healthcare disparities. The risk of LC-19 subsets varies depending on the intensity of the initial COVID-19 illness and between the sexes [98]. According to the Kaiser Family Foundation report, race and ethnicity had a role to play in infections, with non-white patients more affected by COVID-19 and a higher rate of morbidity [99]. The unequal rates among whites and non-whites of diagnosis and treatment for LC-19 indicate the inequities in the healthcare system [100]. Whether this numerical discrepancy is real or just a consequence of the reduced availability of resources for testing and diagnosis is unclear.

### 3.6. Empirical Referents

A concerted effort by the WHO has resulted in developing a consensus definition of LC-19 using the Delphi process. Delphi is a consensus-seeking exercise with an iterative survey involving internal and external experts, patients, and other stakeholders (researchers, external experts, WHO staff, advocacy groups, policymakers, health and disability insurance providers, and media) [35,36,101]. Through collaboration between a panel of experts, the Delphi protocol serves as a communication technique to develop a systemic definition of a concept, including a consensus on variables and values. A detailed questionnaire with 45 items was evaluated in two Delphi process rounds to create a final consensus definition. A clinical case definition was established using pre-defined thresholds and further refined to include values with borderline significance. The wording was adjusted in an iterative process with patients and patient-researchers [35].

Due to the substantial number of patients affected by LC-19, efforts have been made to alleviate the suffering associated. Therefore, LC-19 is now being considered a disability under the Americans with Disabilities Act (ADA), Section 504, and Section 1557 US-HHS, 2021) [101].

### 3.7. Definition of the Concept

This concept analysis provides an improved definition of LC-19 by connecting the physical symptomology with previously under-addressed factors. On a physiological level, Long COVID-19 (LC-19) is a condition characterized by new, returning, or ongoing symptoms affecting one or more organ systems following the acute phase of a COVID-19 infection. Symptoms cannot be attributed to any pre-existing or alternative diagnosis, as clinicians rule out nonspecific conditions. The physical symptoms and related disabilities are further compounded by significant mental, psychological, emotional, and social impacts experienced by the individual. Thus, the term LC-19 encompasses this condition’s full range of physical, mental, psychological, economic, and social effects. Irrespective of whether Long COVID symptomatology arises from a common pathophysiological mechanism or represents a heterogeneous spectrum of post-viral sequelae, the resulting morbidity is legitimate and substantial [102].

## 4. Impact of the Findings

Knowing the risk of LC-19 features can help plan the availability of relevant healthcare services. Compared to other, more generalized post-viral syndromes, the risk of LC-19 correlates with a COVID-19 infection, suggesting a direct cause-and-effect relationship. This fact may help in developing and tailoring effective treatments against LC-19. Most patients have features of LC-19 in the 3- to 6-month period, and the symptoms in the first three months may help identify those at the most significant risk. One major impact of defining LC-19 as a concept would help reassign social services appropriately according to need, improve social benefits like unemployment and disability, and coordinate methods of improving the isolation and quality of life of those affected.

A clear definition ensures robust estimates of the incidence and co-occurrence of LC-19 features, their relationship to age, gender, or severity of infection, and the extent to which they are specific to symptomatic and pathological details of COVID-19 [103]. As recently described in the NICE (National Institute for Health Care and Excellence) guideline on managing the impact of COVID-19, researchers and healthcare workers should focus efforts on understanding the risk factors and their effect on the development of LC-19 [104]. Further scrutiny into the connection with mental health conditions and their relation to LC-19 would help inform self-questionnaires to increase screening of self-reported symptoms to reduce false-positive diagnoses [105]. These analyses provide comprehensive knowledge of the correlation between pre-existing conditions, co-morbidities, symptoms, and progression of LC-19, which can be used to inform healthcare workers to help identify high-risk populations and develop mitigation strategies [70,106]. It is important to note that as research continues and more strains emerge, the LC-19 definition may have to adapt to encompass new knowledge and address new symptomology.

## 5. Conclusions/Discussion

As outlined in Table 2, the vast range of symptoms of LC-19 makes diagnosis challenging and frustrating for patients and clinicians. Further refinement of diagnostic definitions and methodology is essential to create systemic and inclusive criteria, enabling more consistent identification of LC-19 to aid healthcare personnel. Due to the rapid global spread of the pandemic and worldwide exposure, distinguishing LC-19 from other chronic or post-infectious conditions has become increasingly complex [107,108].

One notable overlap is myalgic encephalomyelitis/chronic fatigue syndrome (ME/CFS), which overlaps with LC-19 in terms of complexity, time frame, and some symptomology [109,110]. Like LC-19, ME/CFS has been seen to affect a broad array of populations, with a predominance of women being affected over men [111]. A systematic review comparing symptoms of LC-19 and ME/CFS suggests many similarities between the two conditions, showing that “out of 29 listed ME/CFS symptoms, all but four were reported in at least one long COVID study [112].” Historically, both conditions have suffered from clinical skepticism, being noted as psychological or quickly dismissed. For CFS, earlier termed the “Yuppie Flu,” symptoms were typically seen within a younger population and misconstrued as avoiding work/responsibilities. While LC-19 has gained more legitimacy in the medical field due to the clear link to a known viral infection, it still shares the potential burden of being misunderstood and socially stigmatized [113].

Establishing a more precise definition of LC-19 is imperative to distinguish it from other latent virus reactivation cases or post-viral syndromes and affirm the legitimacy of patient experiences. Whether the etiology of symptoms is common or heterogeneous across patients, it is imperative to recognize that the associated suffering is both real and substantial. Accordingly, it must be addressed through a multidisciplinary approach encompassing clinical, psychological, and social interventions. Several organizations and societies have worked to define LC-19 based on the collection of symptoms, but more consensus is needed. The definitions must evolve as research progresses to incorporate new knowledge surrounding LC-19’s pathophysiology, duration, and long-term impact. Public awareness and media attention can also aid in disseminating accurate information, normalizing the condition, and reducing stigma, playing a positive role in coping with the effects of LC-19. Therefore, continued efforts must be conducted to define LC-19 as a distinct condition, especially compared to related chronic illnesses. A formal acknowledgment of the condition, its defining attributes, and symptoms will go a long way in legitimizing the diagnosis to reduce the social stigma and barriers to healthcare and social care [113,114].

Looking forward, improving diagnostic precision for LC-19 will require considering numerous factors. When diagnosing LC-19, clinicians must evaluate a patient’s history to rule out other or unrelated conditions. Initially, this process utilized self-reporting questionnaires, but clinical measures have been the primary tool for diagnosing LC-19 as the condition progressed. Further strengthening of the alignment between subjective self-reporting measures and clinical outcomes could increase diagnostic accuracy and reduce patient bias [105]. Emerging research has identified promising definitive diagnostic tools, including a simple blood-based test that distinguishes LC-19 patients from those who have fully recovered, based on differential gene expression [115]. Additionally, new diagnostic scales are being developed to assess a patient’s risk for LC-19 development, further aiding clinicians in early identification and intervention [116,117,118]. Long-term treatment of LC-19 will necessitate coordinated multidisciplinary teams of clinicians across cardiology, pulmonology, neurology, rehabilitation medicine and mental health services [106]. Tools such as work ability indexes, patient-reported outcome measures, and continual clinical assessments will be beneficial in monitoring the progression and guiding rehabilitation strategies of LC-19 patients.

In conclusion, LC-19 represents a complex and pressing public health challenge that has emerged from the COVID-19 pandemic. Its diverse symptomology, unpredictable timeline, and complex pathophysiology demand a continual focus from clinicians as a post-viral condition. As research indicates, LC-19 is not a monolithic condition, but a spectrum driven by various biological, immunological, and psychosocial mechanisms. The future of LC-19 care should prioritize early identification through standardized diagnostics, investment into biomarker research, and the development of targeted, multidisciplinary treatment strategies. Additional consideration and research must occur as new variants emerge. Overall, long COVID-19 remains a dynamic disease with evolving diagnostic and therapeutic approaches, requiring deeper investigation and understanding of its long-term effects.

## Figures and Tables

**Table 1:** Common symptoms that can be associated with LC-19.

Symptoms of LC-19	
NEUROLOGICAL [42]	Cognitive Impairment, Neuropsychiatric Symptoms, Depression, Anxiety
CARDIOVASCULAR [43]	Cardiomyopathy, Chest Pain, Thrombophilia, Tachycardia, Palpitations
RESPIRATORY [44]	Pulmonary Dysfunction, Lung Fibrosis, Cough, Shortness of Breath
IMMUNE RESPONSE [45]	Inflammation, Impaired Tissue Repair, Fever
MUSCULAR [46]	Fatigue, Myalgia

**Table 2:** Genes associated with LC-19 pathogenesis in the literature, OMIM gene reference ID, and their symptoms.

Genes Associated With Symptoms in LC-19		
GENE	OMIM ID	SYMPTOM
*FOXP4* [56,57]	608924	Pulmonary Dysfunction
*TLR4* [58]	603030	Cognitive Impairment
*IL6* [59]	147620	Fatigue, Inflammation, Neuropsychiatric Symptoms
*TNF-α* [60]	191160	Fatigue, Inflammation
*VEGF-A* [59]	192240	Cardiomyopathy, Pulmonary Dysfunction
*FOXO1* [60]	136533	Cardiomyopathy
*CXCR4* [60]	162643	Cardiomyopathy
*SMAD4* [60]	600993	Cardiomyopathy
*INF-γ* [61,62]	147569	Thrombophilia, Inflammation, Impaired Tissue Repair
*ACE2* [59]	300335	Impaired Tissue Repair
*TMPRSS2* [59]	602060	Immune Response
*IL1* [59]	147720	Inflammation, Depression
*CCL11* [59]	601156	Cognitive Impairment
*NFL* [59,62]	162280	Cognitive Impairment, Fatigue
*GFAP* [59,62]	137780	Cognitive Impairment
*GSTP1AB* [63]	134660	Cognitive Impairment, Myalgia
*GSTO1* [63]	605482	Cognitive Impairment, Myalgia
*RTEL1* [64]	608833	Disease severity, Higher liver function indices, Lung Fibrosis
*NLPR3* [65]	606416	Major Depressive Disorder, Fatigue, Cognitive Impairment, Anxiety

## Data Availability

The data are available upon reasonable request.
